# Longitudinal Analysis of Neuraminidase and Hemagglutinin Antibodies to Influenza A Viruses after Immunization with Seasonal Inactivated Influenza Vaccines

**DOI:** 10.3390/vaccines11111731

**Published:** 2023-11-20

**Authors:** Mariia V. Sergeeva, Ekaterina A. Romanovskaya-Romanko, Vera Z. Krivitskaya, Polina A. Kudar, Nadezhda N. Petkova, Kira S. Kudria, Dmitry A. Lioznov, Marina A. Stukova, Yulia A. Desheva

**Affiliations:** 1 Smorodintsev Research Institute of Influenza, Ministry of Health of the Russian Federation, 197022 Saint Petersburg, Russia; mari.v.sergeeva@gmail.com (M.V.S.); romromka@yandex.ru (E.A.R.-R.); vera.krivitskaya@influenza.spb.ru (V.Z.K.); kira336@yandex.ru (K.S.K.); dlioznov@yandex.ru (D.A.L.); marina.stukova@influenza.spb.ru (M.A.S.); 2 ‘Institute of Experimental Medicine’, 197022 Saint Petersburg, Russia; polina6226@mail.ru (P.A.K.); pn.nadezhda@yandex.ru (N.N.P.)

**Keywords:** influenza, vaccines, antibodies, neuraminidase-inhibition, persistence

## Abstract

Neuraminidase (NA)-based immunity could reduce the harmful impact of novel antigenic variants of influenza viruses. The detection of neuraminidase-inhibiting (NI) antibodies in parallel with anti-hemagglutinin (HA) antibodies may enhance research on the immunogenicity and duration of antibody responses to influenza vaccines. To assess anti-NA antibodies after vaccination with seasonal inactivated influenza vaccines, we used the enzyme-linked lectin assay, and anti-HA antibodies were detected in the hemagglutination inhibition assay. The dynamics of the anti-NA antibody response differed depending on the virus subtype: antibodies to A/H3N2 virus neuraminidase increased later than antibodies to A/H1N1pdm09 subtype neuraminidase and persisted longer. In contrast to HA antibodies, the fold increase in antibody titers to NA after vaccination poorly depended on the preexisting level. At the same time, NA antibody levels after vaccination directly correlated with titers before vaccination. A difference was found in response to NA antigen between split and subunit-adjuvanted vaccines and in NA functional activity in the vaccine formulations.

## 1. Introduction

Since the start of the COVID-19 pandemic, the World Health Organization (WHO) has reported an unprecedented decrease in global influenza virus circulation. Since April 2020, in most countries, influenza circulation has been significantly reduced due to travel restrictions, social distancing, the closure of workplaces and schools, enhanced hygiene, and the use of personal protective equipment. At the same time, the lack of influenza circulation may reduce herd immunity and increase the susceptibility of the population to future epidemics. In this regard, influenza vaccination is of great importance [1].

Various approaches are used to prevent and reduce the incidence of seasonal and pandemic influenza; among them, vaccination is the most effective. Currently used seasonal influenza vaccines are usually available in trivalent or quadrivalent forms, including two subtypes of influenza A viruses—A/H1N1 and A/H3N2—and influenza B viruses of one or two antigenic lineages, B/Victoria and B/Yamagata, according to the WHO’s annual vaccine recommendations. The production of quadrivalent vaccines reduces the risk of vaccine mismatch with epidemic strains [2]. Nevertheless, recently, for the first time in 35 years, there has been an observed disappearance of influenza B/Yamagata antigenic lineage viruses from the circulation [1].

Split and subunit-inactivated influenza vaccines (IIVs) are mainly used for seasonal influenza prevention [3]. In a split virion vaccine, the viral particles are destroyed with diethyl ether or detergents. Split vaccines contain 15 micrograms of HA antigen for each currently recommended influenza strain (A/H1N1pdm09, A/H3N2, B) [4]. Subunit vaccines contain hemagglutinin (HA) and neuraminidase (NA), which are purified by the exclusion of viral RNP and several internal proteins. Vaccines must comply with WHO safety and efficacy requirements. The production of influenza vaccine in chick embryos is a highly productive and reliable technology that meets the quality criteria established by the WHO and is set out in the guidelines for influenza vaccine production and control [5]. Parenteral split or subunit influenza vaccines induce a strain-specific serum IgG antibody response and can be used to prevent influenza in children as young as 6 months of age, as well as in older people suffering from chronic diseases [6,7]. To enhance the immune response to vaccination, a search is being made for safe adjuvants that can enhance the immune response and increase its rate and duration [8].

Seroconversion of hemagglutination-inhibiting (HI) antibodies represents the “gold-standard” criteria to evaluate the immunogenicity of inactivated influenza vaccines. At the same time, the development of new, improved, and standardized methods for the evaluation of neuraminidase-inhibiting (NI) antibodies is repeatedly emphasized by the WHO [9,10]. In recent years, research on NA-specific antibodies has intensified, and NI-antibodies have been described as possessing cross-reactive and protective properties [11]. NA research can promote a better understanding of how NA properties can help develop new cross-reactive vaccines [12].

Neuraminidase-specific antibodies were shown to limit influenza transmission, decrease the severity of infection, and reduce the occurrence of secondary complications [13,14]. The fact that NI antibodies acquired after infection or prior vaccination demonstrate a broad spectrum associated with a reduction in the likelihood of influenza disease supports the inclusion of NA in next-generation vaccines aimed at better protection against drift variants [15,16]. Despite the recognized importance of NA-reactive antibodies in protection against influenza, the content of NA in seasonal influenza vaccines is currently not regulated or measured by manufacturers [16]. Since NA-based immunity may enhance protection against novel antigenic variants of the influenza virus [17], the study of antibodies to NA may play a role in predicting herd immunity against newly emerging influenza viruses, as well as estimating the protection of seasonal influenza vaccines in cases of vaccine mismatch.

The aim of the study was to evaluate the production of NA-inhibiting (NI) antibodies after immunization with seasonal influenza vaccines, the duration of antibody persistence, and the relationship with other quantitative parameters of the immune response to vaccination.

## 2. Materials and Methods

### 2.1. Vaccines

The study included three seasonal trivalent splits or subunit-adjuvanted IIVs. The strain composition of the vaccines corresponded to the WHO recommendations for the northern hemisphere in the 2018–2019 season: A/Michigan/45/2015 (H1N1)pdm09-like virus; A/Singapore/INFIMH-16-0019/2016 (H3N2)-like virus; and B/Colorado/06/2017-like virus (B/Victoria/2/87 lineage) [18]. All vaccines were produced according to Russian Pharmacopeia from purified egg-grown candidate vaccine viruses. HA content in vaccine formulations was indicated according to a single-radial-immunodiffusion assay; NA content was not indicated. The split vaccine ‘Ultrix’ (produced by FORT Biopharmaceutical Company, Moscow, Russia) contained 15 µg of HA of each strain in a 0.5 mL dose [19]. Subunit vaccine ‘Grippol plus’ (produced by Petrovax Pharm, Moscow, Russia) contained 5 µg of HA of each strain (antigens produced by Abbott Biologicals B.V., Olst, The Netherlands) and 500 µg of Polyoxidonium^®^ adjuvant in a 0.5 mL dose [20]. Another subunit vaccine ‘Sovigripp’ (produced by NPO Microgen, Republic of Bashkortostan, Russia) contained 5 µg of HA of influenza A (H1N1)pdm09 and A (H3N2) strains, 11 µg of influenza B strain, and 500 µg of Sovidon^®^ adjuvant in a 0.5 mL dose [21].

### 2.2. Study Design

Serum samples were obtained within the framework of the serological survey. The study included healthy volunteers >18 years old who had no contradictions to vaccination and who had signed the written informed consent. Vaccination was conducted in a specialized clinic of the Smorodintsev Research Institute of Influenza in an open-label regime. Blood samples were collected at the planned time points, including one point before vaccination (day 0) and five points after vaccination (day 7, day 21, 3 months, 6 months, and 12 months). The study was approved by the Local Ethics Committee of the Smorodintsev Research Institute of Influenza, protocol #131 date 10 October 2018.

### 2.3. Hemagglutination Inhibition Assay

The assay was performed as recommended by the WHO [22], using influenza antigens bearing the same HA as in the vaccine strains. Antigens for the HI reaction were type A influenza viruses (subtypes H1N1pdm09, H3N2), antigenically relevant, avid to antibodies, but resistant to nonspecific hemagglutination inhibitors of human sera, grown in the allantoic cavity of chick embryos, inactivated, and lyophilized (LLC “PPDP”, St Petersburg, Russia). Sera were treated with receptor-destroying enzyme (RDE, Denka Seiken Co., Tokyo, Japan) according to the manufacturer’s instructions. Each sample (tested in duplicate) was 2-fold serially diluted in 96-well U-bottom polymer plates starting from 1:10 and mixed with 4 hemagglutination units (HAU) of influenza antigen. After 1 h of incubation, 0.5% chicken erythrocyte suspension was added. The titer of the HI antibody was calculated as the highest serum dilution that inhibited erythrocyte agglutination. Antibody seroconversion was identified as a four-fold or greater increase in the HI antibody titer in comparison to baseline.

### 2.4. Influenza Viruses

Neuraminidase-specific antibodies were assessed in the enzyme-linked assay (ELLA) using purified reassortant influenza viruses: HA from A/herring gull/Sarma/51c/2006 (H6N1) influenza virus and NA from A/South Africa/3626/13 (H1N1)pdm09 or A/Hong Kong/4801/2014 (H3N2) strains. Viruses were obtained from the collection of the Virology Department, Institute of Experimental Medicine, Saint Petersburg, Russian Federation. The viruses were propagated in 10-day-old chicken embryos. The virus-containing allantois fluid was first clarified by centrifugation at 3000 rpm for 20 min. Then the virus was pelleted by ultracentrifugation at 17,000 rpm for 3 h (rotor Type 19, Beckman Optima TM L-100 XP Ultracentrifuge, Brea, CA, USA). The virus pellet was resuspended in ice-cold calcium-borate buffer pH 7.2 and subsequently purified through a sucrose step gradient (30–60%) by ultracentrifugation at 20,000 rpm for 2.5 h (rotor SW40Ti, Beckman, Brea, CA, USA). The virus-containing fraction was extracted and concentrated by centrifugation at 20,000 rpm for 2.5 h (SW 40 Ti rotor, Beckman, Brea, CA, USA). The purified, concentrated virus was resuspended and stored in calcium-borate buffer. The antigen concentration was expressed in HAU.

### 2.5. Enzyme-Linked Lectin Assay (ELLA)

NA-inhibiting antibodies were evaluated by ELLA as described previously in heat-inactivated serum samples (56 °C for 30 min) [23]. In brief, 96-well ELISA plates (Greiner Bio-One, Kremsmünster, Austria) were coated with 50 μg/mL fetuin (Cat. No. F3004, Sigma-Aldrich, St. Louis, MO, USA) overnight at 2–8 °C. Serum samples serially pre-diluted in PBS-1% BSA (Cat. No. A7030, Sigma-Aldrich, St. Louis, MO, USA) were incubated with an equal volume of pre-diluted virus for 30 min at 37 °C. The content of influenza viruses was 128–256 HAU/0.1 mL, which gave OD450 in the range of 0.4–0.6. After incubation, 100 μL of the mixtures was applied to the fetuin-coated wells. After incubation for 1 h at 37 °C, the plates were washed, and NA activity was assessed by incubating with peroxidase-labeled peanut lectin (2.5 μg/mL, Cat. No. L7759, Sigma-Aldrich, St. Louis, MO, USA) for 1 h, RT, followed by a washing step. Then, TMB substrate (BD Biosciences, San Diego, CA, USA) was added for 5 min, and the reaction was stopped by 1N sulfuric acid. Optical density (450 nm) was measured by an ELx800 microplate reader (Bio-Tek Instruments Inc., Winooski, VT, USA). The titer of NI antibodies was determined as a serum dilution, which provided a 50% decrease in optical density in comparison with the virus control wells. A two-fold increase in NI antibody titer after vaccination was considered antibody seroconversion.

### 2.6. NA Activity of Influenza Viruses and Influenza Vaccines

The NA activity of influenza viruses and influenza vaccines was measured by ELLA using the high molecular substrate fetuin (Sigma-Aldrich, St Lous, MO, USA). Virus or vaccine 2-fold dilutions were prepared in phosphate-buffered saline containing bovine serum albumin (PBS-BSA) starting from 256 HAU in triplicate.

### 2.7. Statistical Analysis

Statistical data analysis and visualization were performed using MS Excel 2016, RStudio 2022.12.0, and GraphPad Prizm 8.4.3. A number of group descriptive statistics, including proportion (%), mean, median, interquartile range (IQR), and geometric mean, were calculated. The confidence interval for the geometric mean was calculated by transforming the titers to logarithms, then calculating the 95% CI, and then taking the antilog of the interval limits. The confidence interval (95%CI) for proportion was calculated by the Clopper–Pearson method. Statistical analysis was exploratory and was performed post hoc; particular statistical tests are indicated in the text. For statistical analysis, the logarithmic values of antibody titers were used. The difference was considered statistically significant at a *p*-value of <0.05. Regression analyses were performed with the ‘glm’ function from the ‘stats’ package in RStudio using the binomial family model. The correlation matrix and heatmap were generated using Spearman correlation analysis for multiple variables as implemented in Prism 8.4.3 (GraphPad, Boston, MA, USA).

### 2.8. Study Limitations

The present study included participants 18 years of age and older with an unknown history of previous vaccinations and infections, both seropositive and seronegative. The study was open-label, and no special inclusion criteria were used except for eligibility for vaccination, so the study cohort does not represent any specific population. The study aimed to analyze the humoral immune response to HA and NA influenza antigens and did not include analyses of cell-mediated responses after vaccination or the protection rate in vaccinated groups. The study focuses on antibody analysis at one time point before vaccination and five time points within a year after vaccination with the inactivated influenza vaccine, and presents the data from 73 subjects with a complete specimen set.

## 3. Results

### 3.1. Antibody Levels throughout the Year after Vaccination

Assessment of NA and HA inhibiting antibodies was performed in serum samples obtained from 73 healthy individuals (aged from 20 to 87) before and throughout the year after influenza vaccination. Among the study group, 46 participants were immunized with a subunit-adjuvanted vaccine, and 27 volunteers were immunized using a split vaccine. The main demographic characteristics of the study population are presented in Table 1.

The majority of subjects had a previously unknown history of influenza vaccination. The overall level of seropositivity to HA and NA antigens at the moment of vaccination varied from middle (37% against N2) to high (85% against H1pdm09).

**Figure 1:**
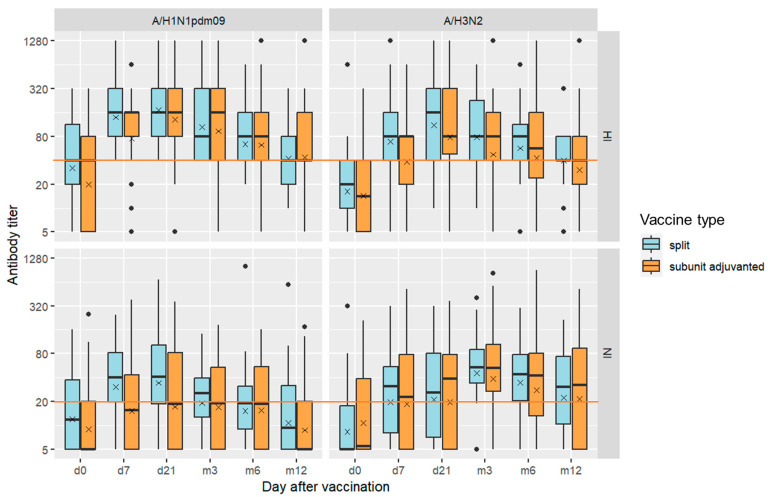
Antibody dynamics throughout 1 year after vaccination with subunit-adjuvanted vaccine (*n* = 46) or split vaccine (*n* = 27). Data are represented by a box plot diagram with a solid black line at the median titer. Cross symbol indicates the group geometric mean titer (GMT). Red lines indicate the conventional antibody level threshold adopted in this study: 1:40 for HI antibodies and 1:20 for NI antibodies.

The levels of NI and HI antibodies throughout the year after vaccination showed expected and similar dynamics. With the exception of anti-N2 antibodies, the rise of geometric mean antibody titers (GMTs) was detected already at day 7 after vaccination, continued until day 21, and fell down 3 months after vaccination (Figure 1, Supplementary Table S1). Slower dynamics was observed for N2-specific antibodies, with the maximum GMT observed at 3 months after vaccination. Overall NI antibody titers before and after vaccination using both split IIV and adjuvanted IIVs were noticeably lower than the HI antibody titers.

Compared to baseline, a statistically significant increase in the average HI and NI titers was observed at almost all time points starting from day 7 after vaccination for both split and subunit-adjuvanted vaccine groups. All *p*-values are given in the supplementary data (Supplementary Table S2). A year after immunization with the split IIV, a statistically significant increase in antibody titers to both antigens of the A/H3N2 influenza virus still persisted. A year after immunization with adjuvanted IIVs, the level of antibodies significantly differed from the pre-vaccination level only for H1-specific HI antibodies and NI antibodies to N2 (Supplementary Table S2).

Exploratory analysis of variance did not find statistically significant differences between the split and subunit-adjuvant vaccine groups in HI and NI antibody titers at any of the time points. Stratification by high or low baseline antibody titers (<1:40 for HI antibodies and <1:20 for NI antibodies) also showed no statistically significant difference between split and subunit-adjuvanted vaccine groups (Supplementary Figures S1 and S2).

### 3.2. Antibody Seroconversion Rates after Vaccination

To compare HA- and NA-specific antibody responses, we compared the seroconversion rates of HI and NI antibodies on day 21 after vaccination. Split IIVs caused significantly more NI antibody conversions to the A/H1N1 influenza virus compared to adjuvanted IIVs (Table 2).

**Figure 2:**
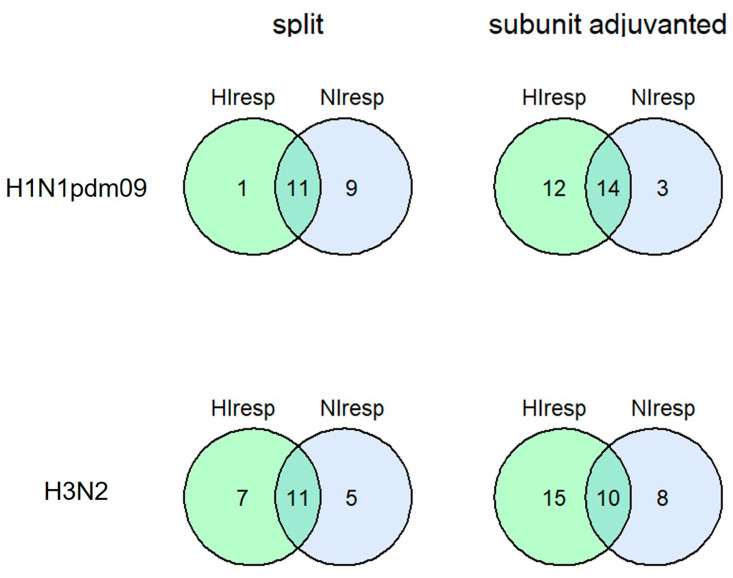
Combined seroconversions of HI and NI antibodies on day 21 after vaccination with split or subunit-adjuvanted IIVs presented by Venn’s diagrams. Numbers in circles present absolute number or responders in one or both tests. The total number of participants was 27 in the split vaccine group and 46 in the subunit-adjuvanted vaccine group. The number of non-responders to both antigens is not shown on Venn’s diagram.

The same trend was observed for A/H3N2 virus NI antibodies; however, the difference was not statistically significant. It is worth noting that both vaccines met the Committee for Proprietary Medicinal Products (CPMP) criteria on the number of HI antibody seroconversions, which exceeded the 40% threshold [24]. Regression analyses performed for the probability of a positive seroconversion outcome confirmed the statistically significant impact of the vaccine type factor on the response to N1 neuraminidase and showed no impact of age, sex, or initial antibody titer (Appendix A Table A1). To demonstrate the neutralization properties of the sera, we used a microneutralization assay. A strong correlation between HI and MNA antibody titers and the same dynamics of antibodies after vaccination are shown (Supplementary Figure S3). The combined seroconversions to HA and NA were observed on average in half of the participants (Figure 2).

The distribution of the numbers for participants who seroconverted for all four components (H1-HA, N1-NA, H3-HA, and N2-NA) and seroconverted for three of the four components is presented in Supplementary Figure S4.

**Figure 3:**
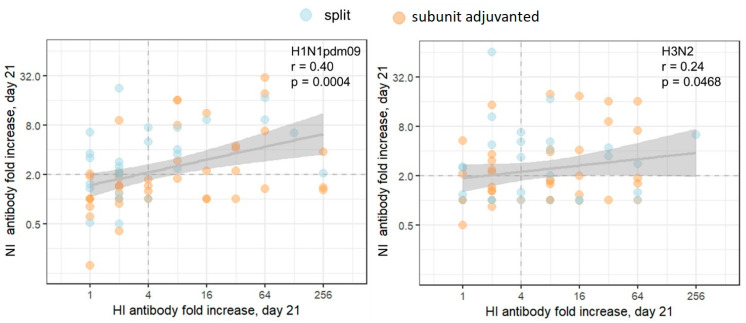
Correlation between post-vaccination HI and NI antibody fold increases. Each dot represents one participant. The results of the Pearson correlation test applied to logarithmic values are presented on graphs. The population in the analyses included all participants, regardless of the vaccine type. The shaded area represents the 95% CI for the regression line. The dashed lines mark the conventional seroconversion threshold (4-fold increase in HI antibody titer and 2-fold increase in NI antibody titer).

Figure 3 represents the correlation analysis of post-vaccination HI and NI antibody titers fold-increase for all subjects in the cohort, including all study participants, both seronegative and seropositive, on day 21 after immunization. The total number of seropositive/negative participants is presented in Table 1. A poor to moderate correlation between HI and NI post-vaccination antibody titer fold increase (Figure 3) assumes some degree of independence in the response to different antigens of the same virus.

### 3.3. Preexisting Immunity Impact and Correlation Analysis of HI and NI Antibody Titers

**Figure 4:**
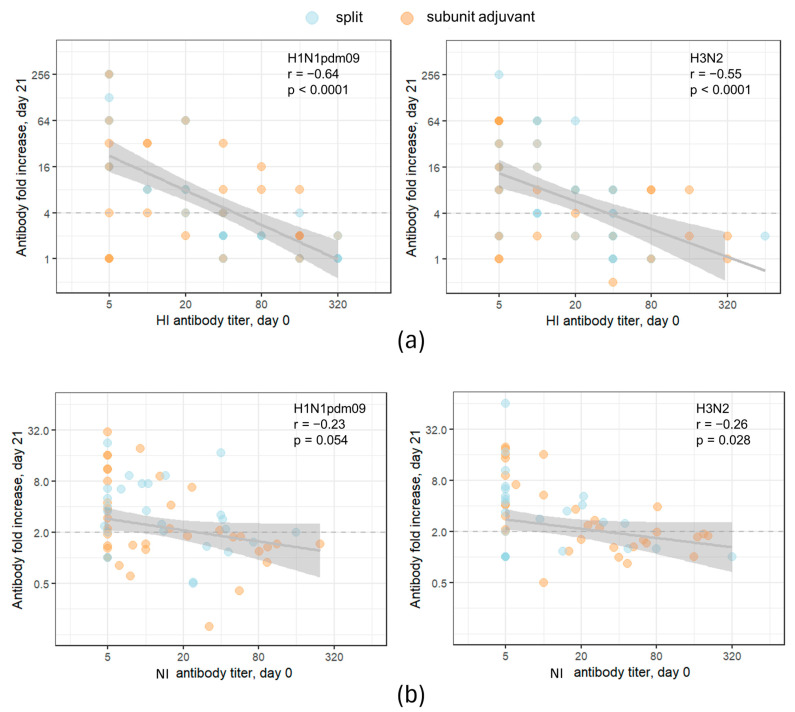
Post-vaccination antibody response dependence on the preexisting antibody level. (**a**) HI antibodies; (**b**) NI antibodies. Each dot represents one participant. The results of the Pearson correlation test applied to logarithmic values are presented on graphs. The population in the analyses included all participants, regardless of the vaccine type. The shaded area represents the 95% CI for the regression line. Participants with an antibody titer of < 10 are considered seronegative. The dashed lines mark the conventional seroconversion threshold (4-fold increase in HI antibody titer and 2-fold increase in NI antibody titer).

Next, we analyzed the dependence of the response to vaccination on the preexisting level of HI and NI antibodies. Figure 4 represents the dependence of the post-vaccination antibody fold increase on the baseline (preexisting) antibody level for all subjects in the cohort. On the left side of the graph X axis are participants with low baseline titers (including seronegative), and on the right side are those with high baseline titers (seropositive). This type of analysis does not presume grouping by the baseline titer. As expected, the post-vaccination HI antibody fold increase is strongly negatively correlated with initial antibody titers (Figure 4a). A poor negative correlation was found between post-vaccination NI antibody titer fold increase and preexisting NI antibody level (Figure 4b).

**Figure 5:**
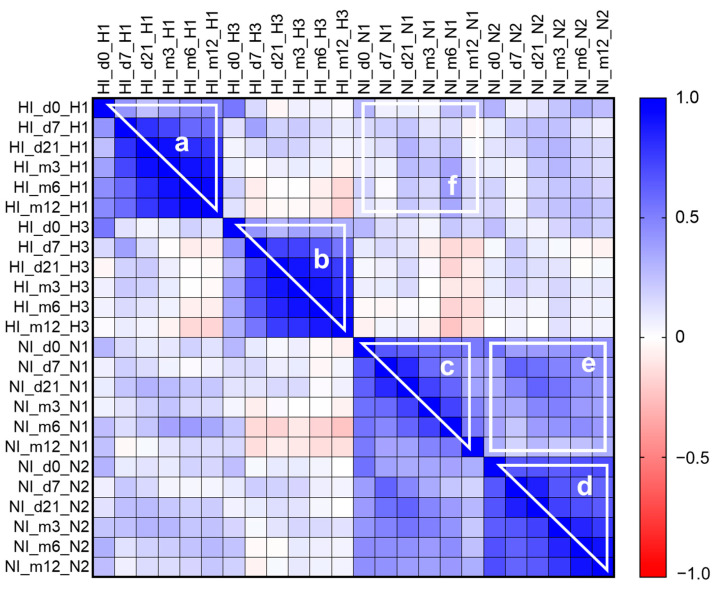
Correlation matrix between HI and NI antibody titers throughout the year after vaccination. The population in the analyses included all participants, regardless of the vaccine type. The heatmap represents the level of correlation between each pair of listed parameters. The color scale shows the Spearman correlation coefficient for positive (blue) or negative (red) correlation; a white color means no correlation. The lower left and upper right corners present the same data (the map is symmetrical about the diagonal), and the squares on the diagonal should not be considered as they represent the “self-correlation.” White-outlined areas can be interpreted as follows: (**a**,**b**) show a positive correlation of anti-HA antibody titers early and late after vaccination, except for the baseline titer for A/H1N1pdm09 and A/H3N2 viruses, respectively; (**c**,**d**) show positive correlation of anti-NA antibody titers early and late after vaccination, including the baseline titer for A/H1N1pdm09 and A/H3N2 viruses, respectively; (**e**) shows a low positive correlation between anti-NA antibody titers against two influenza A subtypes; (**f**) shows an absence of correlation between anti-HA and anti-NA antibody titers.

Further, we performed the rough correlation matrix analyses of HI and NI antibody titers to A/H1N1pdm09 and A/H3N2 influenza viruses before, shortly after vaccination, and throughout a year (Figure 5). Analysis of HI antibody titers has shown a strong positive correlation between titers on days 7 and 21, and 3, 6, and 12 months after vaccination, and a much weaker correlation with day 0 (Figure 5a,b), which can be interpreted as the higher the antibody titer after vaccination, the higher it will be during the whole year independently of the titer before vaccination. On the contrary, the titer of NI antibodies at day 0 was more strongly correlated with titers at any time point after vaccination (Figure 5c,d), which can be interpreted as the higher the initial NI antibody titer before vaccination, the higher it will be during the year after vaccination. A positive correlation was also found between anti-NA antibody titers against two influenza A subtypes (Figure 5e) and almost no correlation between anti-HA and anti-NA antibody titers (Figure 5f).

### 3.4. Neuraminidase Functional Activity in the Vaccine Formulation

**Figure 6:**
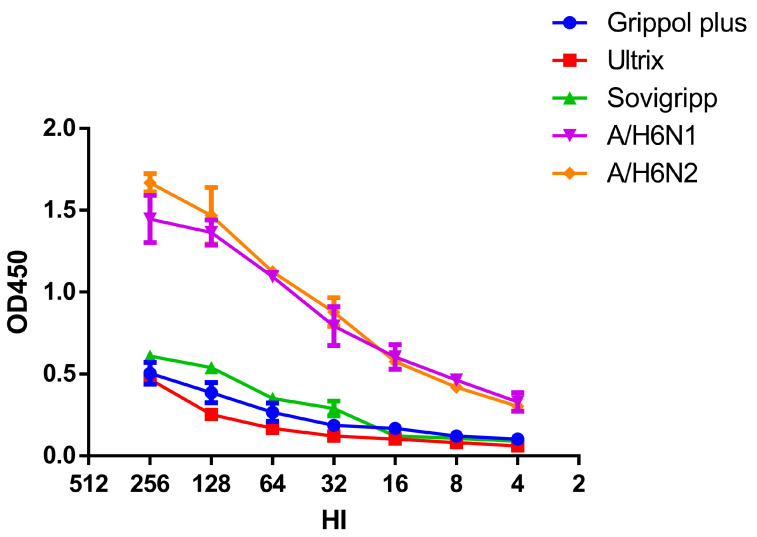
NA enzyme activity of influenza vaccines and H6NX viruses.

Finally, we attempted to evaluate if the NA included in the vaccine preparations was functionally active. The identification of NA ingredients in the formulation of Ultrix and Sovigripp vaccines was previously described in a recently published study [25]. The presence of the NA in the Grippol vaccine is stated in the instructions on medical use of the medicinal product, published on the manufacturer’s official site (https://grippol.ru/en/grippol-kvadrivalent/instruction/, accessed on 6 November 2023). No NA quantity measurement can be found in any of the above publications. In the present study, the sialidase activity of the vaccine preparations was compared with that of the reassortant influenza viruses containing NA of the N1 or N2 subtype (Figure 6).

It turned out that all three vaccines exhibit enzymatic activity, although significantly lower than whole influenza viruses with the same hemagglutination activity (*p* = 0.0009, Kruskal–Wallis test). Therefore, the increase in antibody titers to NA may well be specific rather than non-specific steric hiding of the NA active site by antibodies attached to the highly conserved HA stem region [26].

## 4. Discussion

Vaccination is the most effective and scientifically proven way to prevent influenza infection. Current influenza vaccines have been optimized and standardized specifically to induce high titers of anti-HA antibodies, and the production of anti-NA antibodies is not considered a criterion for immunogenicity. There is evidence that NA can make an additional contribution to the protective efficacy of influenza vaccination [25]; therefore, the study of NI antibody seroconversion may be important for assessing the immunogenicity of influenza vaccines, and this issue has repeatedly attracted the attention of experts from the World Health Organization [9,10]. Since NI antibodies block the enzymatic function of NA [27], this determines the possible mechanisms of their protective action, such as preventing the release of viral progeny from the cell [28]. At the initial stage of the infectious cycle of the influenza virus, NI antibodies can prevent the attachment of HA to sialic receptors by steric shielding [29]. In addition, by attaching to the cell surface, NA antibodies can promote the induction of Fc receptor-mediated effector functions, such as ADCC [30], and mediate the activation of the complement system in the process of complement-dependent cytotoxicity [31].

Animal models have shown that NA-directed antibodies do not completely prevent infection with the influenza virus but reduce the lethality and reproduction of the virus in the lungs [32,33,34]. In humans, NI antibodies are not inferior to HI antibodies in protecting against influenza infection or in preventing the development or reducing the severity of the disease [35]. Natural influenza infection results in high seroconversion rates against both HA and NA [28]. A number of studies have shown that the induction of antibodies towards NA after immunization with seasonal vaccines is substantially reduced when compared to influenza infection [36] and varies widely between manufacturers [35,37,38,39,40]. After a natural influenza infection, the number of NA-reactive B cells was equal to or exceeded HA-reactive B cells, while seasonal influenza vaccines rarely induce NA-reactive B cells [36]. In addition, widely cross-reactive anti-NA antibodies acquired from natural infection did not bind several inactivated vaccines available, suggesting that vaccines lack the NA epitopes targeted by these antibodies [36]. The NA enzymatic activity is an excellent indicator of the native structure and correlates well with immunogenicity [41]. Quite often, it is believed that during the production of IIVs, NA loses its structure and, consequently, its enzymatic activity and immunogenicity [25]. The stability and immunogenicity of NA in a vaccine may depend on the method of virus inactivation and also vary between manufacturers. In our study, it was shown that both split and subunit IIVs exhibited similar enzymatic activity, albeit lower than whole viruses.

The antigenic competition between HA and NA has been postulated, which is mainly due to the larger number of HA proteins found on the virion surface and their amplified presentation to the antigen-recognition cells of the immune system [42]. The amount of NA in vaccines also differs depending on the proportions of HA and NA in each strain. Thus, in the whole virus, the ratio of HA to NA is usually 4–5:1 [43]. Seasonal inactivated vaccines usually contain 15 µg of the HA of each component [38]. In the present study, we used seasonal IIVs: the split vaccine contained 15 μg of HA of each vaccine strain, and both subunit-adjuvant vaccines contained 5 μg of HA. The NA content of the vaccines was unknown. In our study, NI antibody titers after vaccination using both split IIV and subunit-adjuvanted IIVs were noticeably lower than the HI antibody titers, which can partly be explained in terms of the differences in the tests used to detect these antibodies. At the same time, we have demonstrated a statistically significant antibody increase for both HI and NI antibodies to A/H1N1 and A/H3N2 influenza viruses starting from day 7 after vaccination with both the split and subunit-adjuvant IIVs. Besides, the increase in average titers compared with the pre-vaccination level persisted for 12 months in relation to both HI and NI antibodies.

Previously, it was shown that the most pronounced rise in the levels of NA-specific antibodies was observed on the 21st day after immunization with IIV, and after 180 days, the average levels of antibodies to NA were still significantly different from those before vaccination [43]. In our study, the levels of NI antibodies increased already on day 7 after immunization, and it can be noted that they did not differ significantly from those on day 21, while the level of antibodies to HA almost always increased from 7 to 21 days after vaccination (Supplementary Tables S1 and S2).

The weak correlation of immune responses to HA and NA on day 21 post-vaccination shown in our study coincides with previously obtained data [34] and once again emphasizes that NI antibodies can be independent indicators of post-vaccination immunity to influenza. Multivariate analyses performed in earlier studies have shown that serum HI and NI antibodies are independently correlated with resistance to infection and infection-associated disease. Notably, only serum NI antibodies independently predicted a reduction in incidence among infected subjects [44].

A number of studies have focused on the effect of pre-existing antibody levels on the immune response after vaccination. So, during the 2007–2008 influenza season, it was shown that for individuals with high HI or NI titers before vaccination, the inactivated vaccine may not induce antibody increases due to natural immunity and may not provide additional protection and/or immune response [45]. At the same time, it has recently been shown that infection with the A(H3N2) virus prior to vaccination can increase the number and spectrum of A(H3N2)-reactive antibodies induced by vaccination [46]. Our study showed that the formation of HI antibodies after immunization depended on a low pre-vaccination level of these antibodies; no such dependence was found with respect to NI antibodies.

As mentioned above, to detect HI antibodies, we used the same strains of influenza viruses A/H1N1 and A/H3N2 that were included in the vaccine composition in the 2018–2019 flu season [18]. To detect increases in NI antibodies after immunization, we used reassortant viruses A/H6N1 and A/H6N2 containing the NA of viruses isolated earlier—A/South Africa/3626/13 (H1N1)pdm09 and A/Hong Kong/4801/2014(H3N2). Based on the February 2018 report of the Worldwide Influenza Center (https://www.crick.ac.uk/sites/default/files/2018-07/crick_feb2018_report_for_the_web.pdf, accessed on 6 November 2023), it can be concluded that NA of A/H1N1 viruses underwent minimal evolutionary changes from 2013 to 2018, but A/H3N2 viruses circulating since 2016 acquired a N-linked glycosylation site in N2 at position 245 which is close to the enzymatic site and can sterically shield NA-inhibiting antibodies [47,48]. However, in our study, we did not focus on the absolute values of antibody titers but rather on seroconversions and antibody persistence; thus, we believe the abovementioned difference in NA is not significantly influencing the overall conclusions of the study.

In conclusion, it should be said that studying the immunogenicity of NA in seasonal vaccines will help to improve the effectiveness of influenza vaccination. This can be facilitated by studying the properties of NA when selecting candidates for vaccine strains, standardizing the content of NA in vaccine preparations, and optimizing the manufacturing process to preserve the antigenic structure of NA [49]. Adding a known quantity of conformationally correct NA to current seasonal vaccines may improve efficacy and, potentially, cross-reactivity against drifted strains of influenza [16].

## 5. Conclusions

Immunization with inactivated influenza vaccines led to a significant increase in serum anti-NA antibody titers, slowly waning one year after vaccination.

The dynamics of anti-NA antibody titers differed depending on the virus subtype: antibodies to A/H3N2 virus NA increased later than antibodies to subtypes A/H1N1pdm09 and persisted longer. The increase in antibody titers to NA of influenza A viruses after vaccination did not depend on their preexisting level.

The values of NA antibody titers after vaccination directly correlated with titers before vaccination, in contrast to antibodies to HA.

The split vaccine was more immunogenic in relation to the NI antibody seroconversion rate.

## Figures and Tables

**Table 1:** Study population.

Vaccine Type	Split (*n* = 27)	Subunit-Adjuvanted (*n* = 46)
Vaccine (*n*)	Ultrix (*n* = 27)	Sovigripp (*n* =19) Grippol plus (*n* = 27)
Age group		
18–60, *n* (%)	13 (48%)	33 (72%)
>60, *n* (%)	14 (52%)	13 (28%)
Age—years Median (IQR)		
18–60	39 (32–42)	37 (34–48)
>60	65 (61–67)	72 (66–79)
Sex		
Female, *n* (%)	22 (81%)	31 (67%)
Male, *n* (%)	5 (19%)	15 (33%)
Seronegative, HA *		
H1N1pdm09, *n* (%)	4 (15%)	13 (28%)
H3N2, *n* (%)	5 (19%)	18 (39%)
Seronegative, NA **		
H1N1pdm09, *n* (%)	12 (44%)	29 (63%)
H3N2, *n* (%)	18 (67%)	24 (52%)

* Seronegativity to HA was defined as antibody titer < 10 in HI; ** seronegativity to NA was defined as antibody titer <10 in ELLA.

**Table 2:** Percent of seroconversions among vaccinees on day 21 after vaccination (with 95% CI).

Assay	Subtype	Split (*n* = 27)		Subunit-Adjuvanted (*n* = 46)	
		Percent	95%CI	Percent	95%CI
HI test	H1	44	(25–64)	57	(42–72)
	H3	67	(46–84)	54	(39–69)
ELLA	N1	74 *	(54–89)	37 *	(23–53)
	N2	59	(39–77)	39	(25–54)

* *p* = 0.0022, Chi-square test.

## Data Availability

Particular datasets are available on reasonable request to the corresponding author.

## Supplementary Materials

The following supporting information can be downloaded at: https://www.mdpi.com/article/10.3390/vaccines11111731/s1, Table S1: Average level of NI and HI antibodies throughout a year after vaccination with split and subunit adjuvanted influenza vaccines; Supplementary Table S2: Statistical analyses results of within group antibody titer comparison in dependence of time point; Figure S1: Antibody dynamics throughout 1 year after vaccination in the subgroups of participants with initially low specific antibody titers; Figure S2: Antibody dynamics throughout 1 year after vaccination in the subgroups of participants with initially high specific antibody titers; Figure S3: Neutralizing antibody dynamics and correlation with hemagglutination inhibiting antibody; Figure S4: Distribution of the numbers for participants who seroconverted for all four components (H1-HA, N1-NA, H3-HA and N2-NA), and seroconverted for 3/4 components; Supplementary Methods: Microneutralization assay.

## Appendix A

**Table A1:** Regression analyses results. The impact of preexisting factors on the response (seroconversion) to particular antigens in immunized participants on day 21 after vaccination was analyzed by generalized linear model method (binomial family). The parameter analyzed by the model was the chance of response to vaccination, where the seroconversion on day 21 was considered as positive outcome (1) and the lack of seroconversion as negative outcome (0), thus the binomial family was chosen. * *p* < 0.05, ** *p* < 0.01, *** *p* < 0.001.

**Response→**	HI seroconversion		NI seroconversion	
**Factor** **↓**	H1	H3	N1	N2
Titer at day 0	*p* = 0.0009 ***	*p* = 0.0268 *	*p* = 0.0914	*p* = 0.0522
Vaccine type	*p* = 0.7699	*p* = 0.5658	*p* = 0.0031 **	*p* = 0.1097
Age	*p* = 0.5432	*p* = 0.5389	*p* = 0.8303	*p* = 0.7365
Sex	*p* = 0.0511	*p* = 0.8002	*p* = 0.7034	*p* = 0.1209

## References

[1] Dhanasekaran V., Sullivan S., Edwards K.M., Xie R., Khvorov A., Valkenburg S.A., Cowling B.J., Barr I.G. (2022). Human seasonal influenza under COVID-19 and the potential consequences of influenza lineage elimination. Nat. Commun..

[2] Ambrose C.S., Levin M.J. (2012). The rationale for quadrivalent influenza vaccines. Hum. Vaccines Immunother..

[3] Lavanchy D., Osterhaus A.D. (2001). Recommendations for the use of inactivated influenza vaccines and other preventive measures. Vaccine.

[4] Kon T.C., Onu A., Berbecila L., Lupulescu E., Ghiorgisor A., Kersten G.F., Cui Y.-Q., Amorij J.-P., Van der Pol L. (2016). Influenza vaccine manufacturing: Effect of inactivation, splitting and site of manufacturing. Comparison of influenza vaccine production processes. PLoS ONE.

[5] Hendriks J., Holleman M., de Boer O., de Jong P., Luytjes W. (2011). An international technology platform for influenza vaccines. Vaccine.

[6] Iorio A.M., Rivosecchi P., Zei T., Neri M., Merletti L. (1989). Immune response to trivalent inactivated influenza vaccine in young and elderly subjects. Vaccine.

[7] World Health Organization (2000). Influenza vaccines: Recommendations for the use of inactivated influenza vaccines and other preventive measures. Wkly. Epidemiol. Rec. = Relev. Épidémiologiquehebdomadaire.

[8] Tregoning J.S., Russell R.F., Kinnear E. (2018). Adjuvanted influenza vaccines. Hum. Vaccines Immunother..

[9] Bright R.A., Neuzil K.M., Pervikov Y., Palkonyay L. (2009). WHO meeting on the role of neuraminidase in inducing protective immunity against influenza infection, Vilamoura, Portugal, September 14, 2008. Vaccine.

[10] Krammer F., Fouchier R.A.M., Eichelberger M.C., Webby R.J., Shaw-Saliba K., Wan H., Wilson P.C., Compans R.W., Skountzou I., Monto A.S. (2018). NAction! How can neuraminidase-based immunity contribute to better influenza virus vaccines?. mBio.

[11] Giurgea L.T., Morens D.M., Taubenberger J.K., Memoli M.J. (2020). Influenza neuraminidase: A neglected protein and its potential for a better influenza vaccine. Vaccines.

[12] Krammer F., Palese P. (2015). Advances in the development of influenza virus vaccines. Nat. Rev. Drug Discov..

[13] Maier H.E., Nachbagauer R., Kuan G., Ng S., Lopez R., Sanchez N., Stadlbauer D., Gresh L., Schiller A., Rajabhathor A. (2020). Pre-existing antineuraminidase antibodies are associated with shortened duration of influenza A (H1N1) pdm virus shedding and illness in naturally infected adults. Clin. Infect. Dis..

[14] McCullers J.A., Huber V.C. (2012). Correlates of vaccine protection from influenza and its complications. Hum. Vaccines Immunother..

[15] Weiss C.D., Wang W., Lu Y., Billings M., Eick-Cost A., Couzens L., Sanchez J.L., Hawksworth A.W., Seguin P., Myers C.A. (2020). Neutralizing and neuraminidase antibodies correlate with protection against influenza during a late season A/H3N2 outbreak among unvaccinated military recruits. Clin. Infect. Dis..

[16] Wei C.J., Crank M.C., Shiver J., Graham B.S., Mascola J.R., Nabel G.J. (2020). Next-generation influenza vaccines: Opportunities and challenges. Nat. Rev. Drug Discov..

[17] Eichelberger M.C., Monto A.S. (2019). Neuraminidase, the forgotten surface antigen, emerges as an influenza vaccine target for broadened protection. J. Infect. Dis..

[18] World Health Organization (2018). Recommended Composition of Influenza Virus Vaccines for Use in the 2018–2019 Northern Hemisphere Influenza Season. https://www.who.int/publications/m/item/recommended-composition-of-influenza-virus-vaccines-for-use-in-the-2018-2019-northern-hemisphere-influenza-season.

[19] Erofeeva M.K., Nickonorov I.J., Maksakova V.L., Stukova M.A., Konshina O.S., Okhapkina E.A., Voicehovskaya E.M., Korovkin S.A., Melnikhov S.J., Kiselev O.I. (2014). Protective properties of inactivated virosomal influenza vaccine. Procedia Vaccinol..

[20] Talayev V., Zaichenko I., Svetlova M., Matveichev A., Babaykina O., Voronina E., Mironov A. (2020). Low-dose influenza vaccine Grippol Quadrivalent with adjuvant Polyoxidonium induces a T helper-2 mediated humoral immune response and increases NK cell activity. Vaccine.

[21] Erofeeva M.K., Stukova M.A., Shakhlanskaya E.V., Buzitskaya Z.V., Maksakova V.L., Krainova T.I., Pisareva M.M., Popov A.B., Pozdnjakova M.G., Lioznov D.A. (2021). Evaluation of the Preventive Effectiveness of Influenza Vaccines in the Epidemic Season 2019–2020 in St. Petersburg. Epidemiol. Vaccinal Prev..

[22] World Health Organization (2011). Manual for the Laboratory Diagnosis and Virological Surveillance of Influenza. 2.F Serological Diagnosis of Influenza by Haemagglutination Inhibition Testing. https://apps.who.int/iris/handle/10665/44518.

[23] Desheva Y., Smolonogina T., Sychev I., Rekstin A., Ilyushina N., Lugovtsev V., Samsonova A., Go A., Lerner A. (2018). Anti-neuraminidase antibodies against pandemic A/H1N1 influenza viruses in healthy and influenza-infected individuals. PLoS ONE.

[24] Committee for Medicinal Products for Human Use (1997). Note for Guidance on Harmonisation of Requirements for Influenza Vaccines.

[25] Rajendran M., Nachbagauer R., Ermler M.E., Bunduc P., Amanat F., Izikson R., Cox M., Palese P., Eichelberger M., Krammer F. (2017). Analysis of anti-influenza virus neuraminidase antibodies in children, adults, and the elderly by ELISA and enzyme inhibition: Evidence for original antigenic sin. mBio.

[26] Marcelin G., Sandbulte M.R., Webby R.J. (2012). Contribution of antibody production against neuraminidase to the protection afforded by influenza vaccines. Rev. Med. Virol..

[27] Halbherr S.J., Ludersdorfer T.H., Ricklin M., Locher S., Berger Rentsch M., Summerfield A., Zimmer G. (2015). Biological and protective properties of immune sera directed to the influenza virus neuraminidase. J. Virol..

[28] Rajendran M., Krammer F., McMahon M. (2021). The human antibody response to the influenza virus neuraminidase following infection or vaccination. Vaccines.

[29] Su B., Wurtzer S., Rameix-Welti M.A., Dwyer D., van der Werf S., Naffakh N., Clavel F., Labrosse B. (2009). Enhancement of the influenza A hemagglutinin (HA)-mediated cell-cell fusion and virus entry by the viral neuraminidase (NA). PLoS ONE.

[30] Wohlbold T.J., Podolsky K.A., Chromikova V., Kirkpatrick E., Falconieri V., Meade P., Amanat F., Tan J., tenOever B.R., Tan G.S. (2017). Broadly protective murine monoclonal antibodies against influenza B virus target highly conserved neuraminidase epitopes. Nat. Microbiol..

[31] Gao R., Sheng Z., Sreenivasan C.C., Wang D., Li F. (2020). Influenza A virus antibodies with antibody-dependent cellular cytotoxicity function. Viruses.

[32] Pliasas V.C., Menne Z., Aida V., Yin J.H., Naskou M.C., Neasham P.J., North J.F., Wilson D., Horzmann K.A., Jacob J. (2022). A Novel Neuraminidase Virus-Like Particle Vaccine Offers Protection against Heterologous H3N2 Influenza Virus Infection in the Porcine Model. Front. Immunol..

[33] Desheva Y., Petkova N., Losev I., Guzhov D., Go A., Chao Y.C., Tsai C.H. (2023). Establishment of a Pseudovirus Platform for Neuraminidase Inhibiting Antibody Analysis. Int. J. Mol. Sci..

[34] Desheva Y., Losev I., Petkova N., Kudar P., Donina S., Mamontov A., Tsai C.H., Chao Y.C. (2022). Antigenic Characterization of Neuraminidase of Influenza A/H7N9 Viruses Isolated in Different Years. Pharmaceuticals.

[35] Monto A.S., Petrie J.G., Cross R.T., Johnson E., Liu M., Zhong W., Levine M., Katz J.M., Ohmit S.E. (2015). Antibody to Influenza Virus Neuraminidase: An Independent Correlate of Protection. J. Infect. Dis..

[36] Chen Y.Q., Wohlbold T.J., Zheng N.Y., Huang M., Huang Y., Neu K.E., Lee J., Wan H., Rojas K.T., Kirkpatrick E. (2018). Influenza Infection in Humans Induces Broadly Cross-Reactive and Protective Neuraminidase-Reactive Antibodies. Cell.

[37] Powers D.C., Kilbourne E.D., Johansson B.E. (1996). Neuraminidase-Specific Antibody Responses to Inactivated Influenza Virus Vaccine in Young and Elderly Adults. Clin. Diagn. Lab. Immunol..

[38] Cate T.R., Rayford Y., Niño D., Winokur P., Brady R., Belshe R., Chen W., Atmar R.L., Couch R.B. (2010). A high dosage influenza vaccine induced significantly more neuraminidase antibody than standard vaccine among elderly subjects. Vaccine.

[39] Couch R.B., Atmar R.L., Keitel W.A., Quarles J.M., Wells J., Arden N., Niño D. (2012). Randomized Comparative Study of the Serum Antihemagglutinin and Antineuraminidase Antibody Responses to Six Licensed Trivalent Influenza Vaccines. Vaccine.

[40] Ito H., Nishimura H., Kisu T., Hagiwara H., Watanabe O., Kadji F.M.N., Sato K., Omiya S., Takashita E., Nobusawa E. (2020). Low Response in Eliciting Neuraminidase Inhibition Activity of Sera among Recipients of a Split, Monovalent Pandemic Influenza Vaccine During the 2009 Pandemic. PLoS ONE.

[41] Sultana I., Yang K., Getie-Kebtie M., Couzens L., Markoff L., Alterman M., Eichelberger M.C. (2014). Stability of neuraminidase in inactivated influenza vaccines. Vaccine.

[42] Shanko A., Shuklina M., Kovaleva A., Zabrodskaya Y., Vidyaeva I., Shaldzhyan A., Fadeev A., Korotkov A., Zaitceva M., Stepanova L. (2020). Comparative immunological study in mice of inactivated influenza vaccines used in the Russian immunization program. Vaccines.

[43] Johansson B.E., Moran T.M., Kilbourne E.D. (1987). Antigen-presenting B cells and helper T cells cooperatively mediate intravirionic antigenic competition between influenza A virus surface glycoproteins. Proc. Natl. Acad. Sci. USA.

[44] Couch R.B., Atmar R.L., Franco L.M., Quarles J.M., Wells J., Arden N., Niño D., Belmont J.W. (2013). Antibody correlates and predictors of immunity to naturally occurring influenza in humans and the importance of antibody to the neuraminidase. J. Infect. Dis..

[45] Gilbert P.B., Fong Y., Juraska M., Carpp L.N., Monto A.S., Martin E.T., Petrie J.G. (2019). HAI and NAI titer correlates of inactivated and live attenuated influenza vaccine efficacy. BMC Infect. Dis..

[46] Auladell M., Phuong H.V.M., Mai L.T.Q., Tseng Y.Y., Carolan L., Wilks S., Thai P.Q., Price D., Duong N.T., Hang N.L.K. (2022). Influenza virus infection history shapes antibody responses to influenza vaccination. Nat. Med..

[47] Wan H., Gao J., Yang H., Yang S., Harvey R., Chen Y.Q., Zheng N.Y., Chang J., Carney P.J., Li X. (2019). The neuraminidase of A (H3N2) influenza viruses circulating since 2016 is antigenically distinct from the A/Hong Kong/4801/2014 vaccine strain. Nat. Microbiol..

[48] Powell H., Pekosz A. (2020). Neuraminidase antigenic drift of H3N2 clade 3c. 2a viruses alters virus replication, enzymatic activity and inhibitory antibody binding. PLoS Pathog..

[49] Creytens S., Pascha M.N., Ballegeer M., Saelens X., de Haan C.A.M. (2021). Influenza neuraminidase characteristics and potential as a vaccine target. Front. Immunol..

