# Supplementary material for: Longitudinal Analysis of Neuraminidase and Hemagglutinin Antibodies to Influenza A Viruses after Immunization with Seasonal Inactivated Influenza Vaccines
Source: Vaccines (Basel). 2023 Nov 20;11(11):1731. doi: 10.3390/vaccines11111731 (PMC10675551; doi:10.3390/vaccines11111731)
Supplement: Supplementary file 1 [file vaccines-11-01731-s001.zip › vaccines-2657851-supplementary.pdf]

Supplementary Material

Supplementary Table S1. Average level of NI and HI antibodies throughout a year after vaccination with split and subunit adjuvanted influenza vaccines (GMT, 95%CI)

Antigen	H1		H3		N1		N2	
Vaccine type	split	subunit adjuvanted	split	subunit adjuvanted	split	subunit adjuvanted	split	subunit adjuvanted
day 0	42 (24 – 73)	27 (18 – 40)	19 (13 – 30)	19 (13 – 29)	14 (9-21)	11 (8-15)	10 (6-16)	14 (10-20)
day 7	152 (103-224)	94 (65-136)	84 (50-142)	49 (33-73)	36 (24-55)	20 (14-28)	25 (15-40)	26 (17-39)
day 21	187 (129-269)	170 (109-265)	130 (79-216)	98 (66-146)	42 (26-67)	22 (15-33)	28 (16-46)	28 (18-42)
month 3	115 (79-167)	117 (79-172)	89 (59-134)	61 (41-91)	26 (16-42)	21 (14-30)	53 (36-79)	50 (34-73)
month 6	70 (49-101)	79 (54-116)	65 (43-98)	54 (37-80)	19 (12-29)	19 (13-26)	41 (27-62)	38 (25-57)
month 12	47 (32-68)	54 (37-77)	46 (31-68)	39 (26-58)	13 (8-22)	11 (8-15)	28 (18-45)	30 (19-45)

Supplementary Table S2. Statistical analysis results of within group antibody titer comparison in dependence of time point

	H1			H3			N1			N2		
	Mean Diff	Summary	Adjusted P Value	Mean Diff	Summary	Adjusted P Value	Mean Diff	Summary	Adjusted P Value	Mean Diff	Summary	Adjusted P Value
split												
d0 vs. d7	-0.54	**	0.003	-0.61	***	0.0008	-0.42	****	<0,0001	-0.39	***	0.0007
d0 vs. d21	-0.64	**	0.0013	-0.80	****	<0,0001	-0.48	****	<0,0001	-0.44	***	0.0004
d0 vs. m3	-0.43	*	0.0178	-0.63	****	<0,0001	-0.27	ns	0.0763	-0.72	****	<0,0001
d0 vs. m6	-0.22	ns	0.5619	-0.50	***	0.0009	-0.13	ns	0.9625	-0.61	****	<0,0001
d0 vs. m12	-0.03	ns	>0,9999	-0.36	*	0.0304	0.02	ns	>0,9999	-0.45	****	<0,0001
d7 vs. d21	-0.09	ns	0.1226	-0.19	*	0.0259	-0.07	ns	0.9855	-0.05	ns	>0,9999
d7 vs. m3	0.12	ns	0.1356	-0.01	ns	>0,9999	0.14	ns	0.8374	-0.33	**	0.0061
d7 vs. m6	0.33	***	0.0003	0.12	ns	0.8858	0.29	*	0.0214	-0.22	ns	0.2761
d7 vs. m12	0.51	****	<0,0001	0.25	ns	0.2476	0.44	**	0.0075	-0.05	ns	>0,9999
d21 vs. m3	0.21	***	0.0004	0.17	**	0.0047	0.21	ns	0.1403	-0.28	ns	0.0549
d21 vs. m6	0.42	****	<0,0001	0.30	**	0.0014	0.36	**	0.006	-0.17	ns	0.4668
d21 vs. m12	0.60	****	<0,0001	0.44	***	0.0001	0.51	**	0.0025	0.00	ns	>0,9999
m3 vs. m6	0.21	****	<0,0001	0.13	ns	0.073	0.15	ns	0.2903	0.12	ns	0.4554
m3 vs. m12	0.40	****	<0,0001	0.27	**	0.0011	0.30	ns	0.2182	0.28	**	0.0026
m6 vs. m12	0.19	****	<0,0001	0.14	ns	0.0698	0.15	ns	0.9767	0.16	**	0.0012
Subunit adjuvanted												
d0 vs. d7	-0.55	****	<0,0001	-0.42	***	0.0004	-0.26	**	0.0014	-0.27	**	0.0086
d0 vs. d21	-0.80	****	<0,0001	-0.70	****	<0,0001	-0.31	***	0.0009	-0.30	***	0.001
d0 vs. m3	-0.64	****	<0,0001	-0.49	****	<0,0001	-0.26	**	0.0033	-0.55	****	<0,0001
d0 vs. m6	-0.47	***	0.0001	-0.44	***	0.0006	-0.24	**	0.0027	-0.43	****	<0,0001
d0 vs. m12	-0.29	*	0.0394	-0.31	ns	0.0658	0.03	ns	>0,9999	-0.33	***	0.0002
d7 vs. d21	-0.25	**	0.0098	-0.28	**	0.0017	-0.06	ns	0.9876	-0.03	ns	>0,9999
d7 vs. m3	-0.08	ns	0.9857	-0.08	ns	0.9829	-0.01	ns	>0,9999	-0.29	**	0.0027
d7 vs. m6	0.08	ns	0.9898	-0.03	ns	>0,9999	0.02	ns	>0,9999	-0.17	ns	0.4139
d7 vs. m12	0.26	**	0.0092	0.11	ns	0.9733	0.28	*	0.0141	-0.06	ns	0.9998
d21 vs. m3	0.17	**	0.0015	0.21	****	<0,0001	0.05	ns	0.9994	-0.25	**	0.001
d21 vs. m6	0.33	****	<0,0001	0.25	****	<0,0001	0.08	ns	0.9885	-0.13	ns	0.6451
d21 vs. m12	0.51	****	<0,0001	0.39	****	<0,0001	0.34	**	0.002	-0.03	ns	>0,9999
m3 vs. m6	0.16	***	0.0002	0.05	ns	0.9938	0.03	ns	>0,9999	0.12	ns	0.1782
m3 vs. m12	0.34	****	<0,0001	0.19	*	0.0454	0.29	***	0.0005	0.22	**	0.0084
m6 vs. m12	0.18	****	<0,0001	0.14	***	0.0007	0.26	***	0.0001	0.10	ns	0.3803

Analyses was performed by two-way repeated measures ANOVA or mixed-effect model in case of missing values as implemented in GraphPad Prizm v. 8.4.3. Presented mean difference and p-value are results of within group comparison of logarithmic titers at indicated time points.

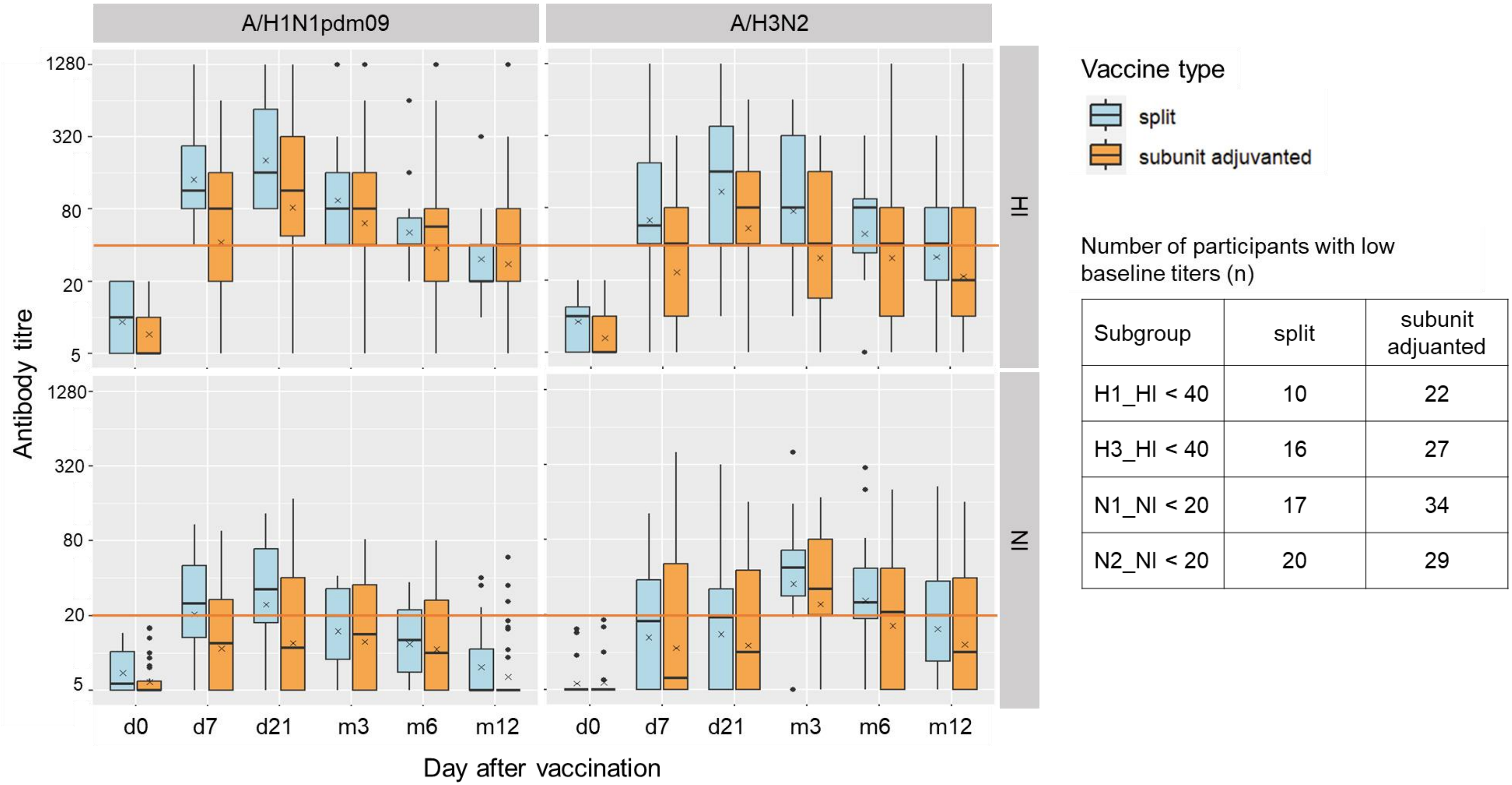

Number of participants with low baseline titers (n)

Subgroup	split	subunit adjuanted
H1_HI < 40	10	22
H3_HI < 40	16	27
N1_NI < 20	17	34
N2_NI < 20	20	29

Supplementary Figure S1. Antibody dynamics throughout one year after vaccination in the subgroups of participants with low baseline antibody titers. Data is represented by box-plot diagram with a solid black line at median titer and a cross at geometric mean titer (GMT). Red lines indicate conventional antibody level threshold adopted in this study: 1:40 for HI antibodies and 1:20 for NI antibodies. Number of participants included in each subgroup are presented in the table on the right.

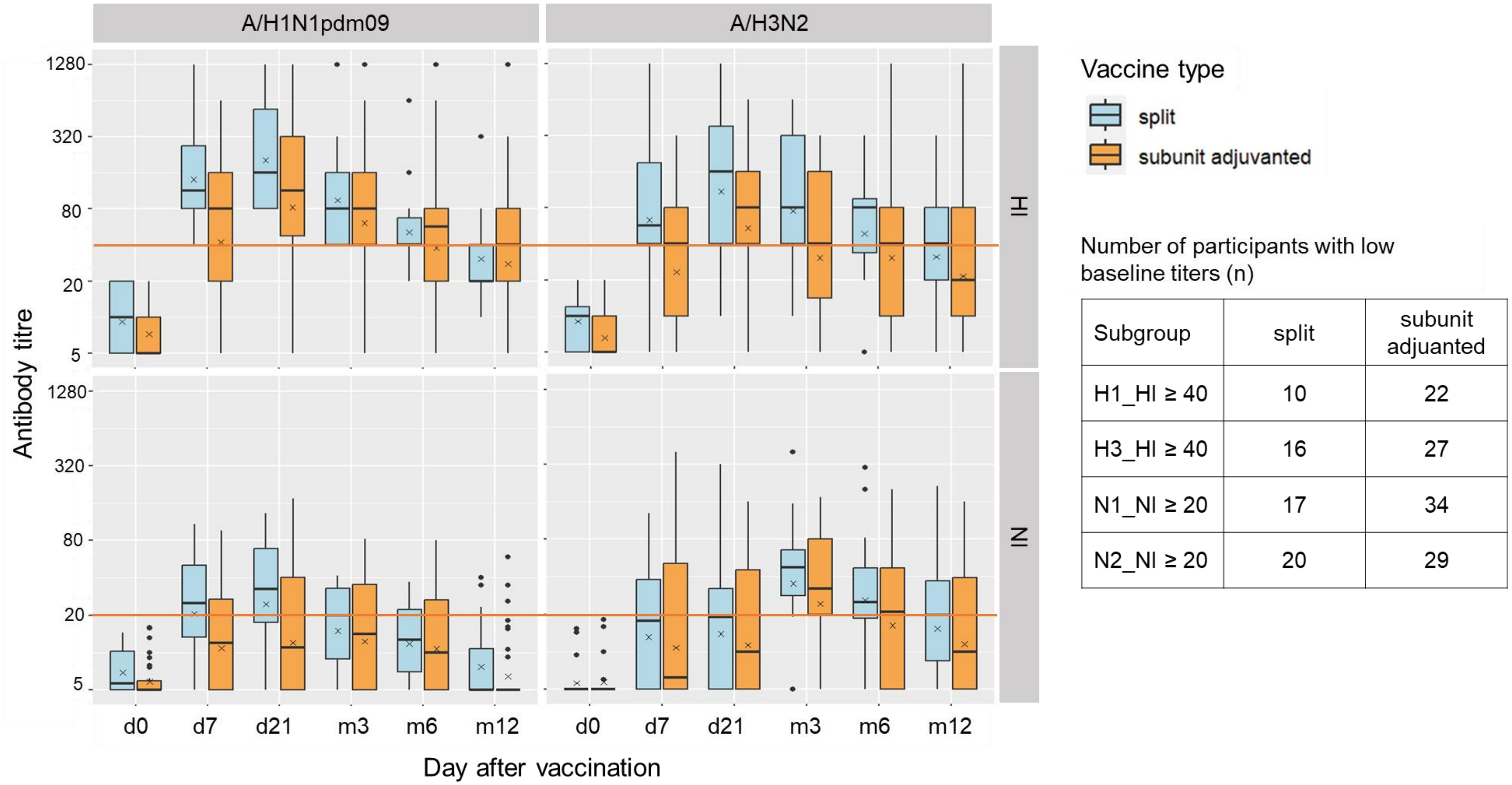

Number of participants with low baseline titers (n)

Subgroup	split	subunit adjuanted
H1_HI ≥ 40	10	22
H3_HI ≥ 40	16	27
N1_NI ≥ 20	17	34
N2_NI ≥ 20	20	29

Supplementary Figure S2. Antibody dynamics throughout one year after vaccination in the subgroups of participants with high baseline antibody titers. Data is represented by box-plot diagram with a solid black line at median titer and a cross at geometric mean titer (GMT). Red lines indicate conventional antibody level threshold adopted in this study: 1:40 for HI antibodies and 1:20 for NI antibodies. Number of participants included in each subgroup are presented in the table on the right.

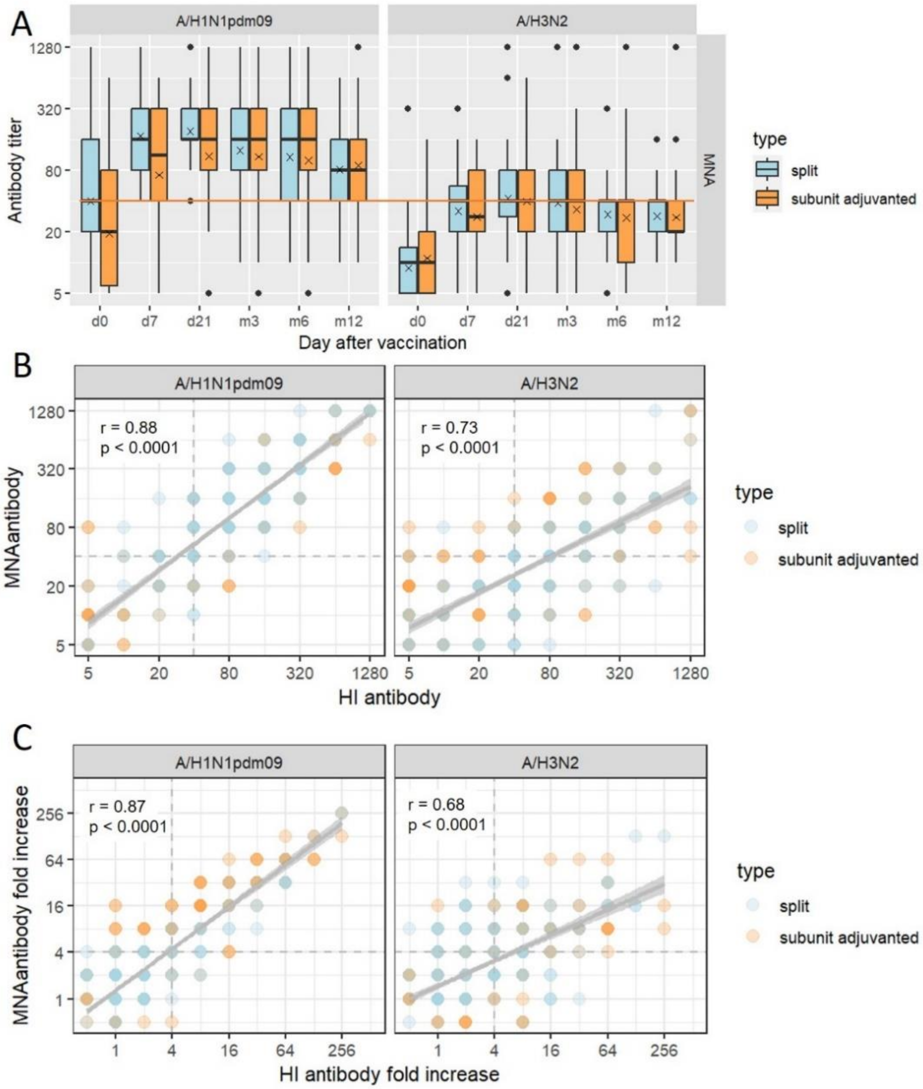

Supplementary Figure S3. Neutralizing antibody dynamics and correlation with hemagglutination inhibiting antibody

(A) Neutralizing antibody throughout 1 year after vaccination. Data is represented by box-plot diagram with a solid black line at median titers and a cross at geometric mean titers (GMT). Red line indicates conventional antibody level threshold (1:40). (B, C) Correlation of HI and MNA data in absolute titers and fold increase values, correspondingly. Each dot represents one participant. Results of Pearson correlation test applied to logarithmic values are presented on graphs. Dotted lines mark the conventional seroconversion threshold (4-fold increase).

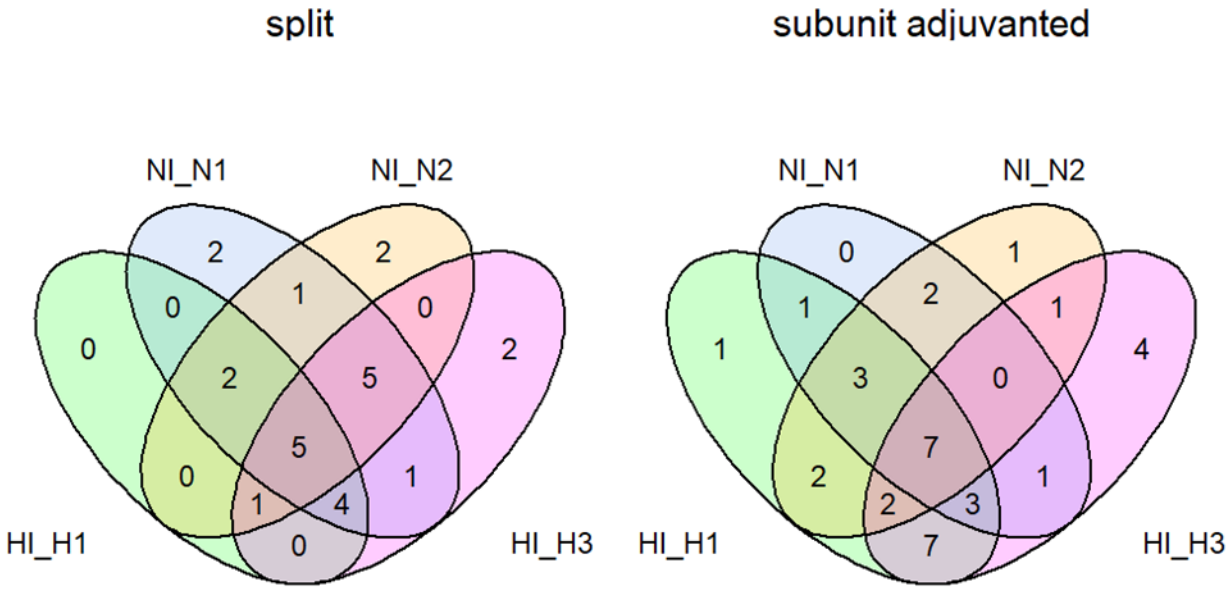

Supplementary Figure S4. Number for participants with seroconversion to all four vaccine components (H1-HA, N1-NA, H3-HA and N2-NA), and combinations, presented in Venn's diagram (number of non-responders to any component not presented).

Supplementary Methods

Microneutralization assay

Microneutralization assay (MNA) was performed as recommended by WHO [22] using live influenza viruses from the collection of the Smorodintsev Research Institute of Influenza. Sera were treated for 16-19 h at 37°C with receptor destroying enzyme (RDE) from Vibrio cholerae NA extract (Denka Seiken Co., Tokyo, Japan) and then heat-inactivated at 56 °C for 30 min. Each serum (tested in duplicate) was 2-fold serially diluted in 96-well U-bottom polymer plates starting from 1:10 and mixed with 100 TCID50 of influenza virus. After 1 h incubation 100 ul of mixture were transferred to MDCK cells (#IRR, FR-58) in 96-well plate and incubated at 37C. After 3-days incubation cells were fixed with 80% acetone (Vekton, Rusia) and stained with HRP-conjugated anti-influenza NP monoclonal antibody 6D11 (produced in the Smorodintsev Research Institute of Influenza). TMB was added to develop colorimetric reaction and optical density at 450 nm was measured by Multiskan MS (Labsystems, Finland). Neutralizing antibody titer was calculated as the reciprocal of the last serum dilution with optical density less than a half of that in virus control wells. A four-fold increase in MNA antibody titer after vaccination was considered as antibody seroconversion.
